# Of Individual Importance to Every One Interested in Dentistry

**Published:** 1880-06

**Authors:** B. M. Wilkerson

**Affiliations:** Junior Editor and Proprietor of “The Independent Practitioner,” 68 N. Charles Street, Baltimore, Md., United States of America


### OF INDIVIDUAL IMPORTANCE TO EVERY ONE INTERESTED IN DENTISTRY.

I am preparing for publication "The Dental Directory of the World," and am exceedingly anxious that the name and address of every dentist should appear correctly in its pages.

This work will contain a general history of dentistry and its individual history in each country, written by authors of acknowledged ability. It will also contain biographical sketches of those who have been the greatest benefactors to the profession; the address of every the dentist in the world; the location and history ef every dental college, with its corps of instructors and their duties; the dental societies, when organized, number of members and times of meeting; also the laws of each State and Country governing the practice of dentistry. It will be of great value to the dental profession as a work of reference.

I most respectfully ask all the dentists in the world; the deans of all dental colleges; the secretaries of all dental societies, and other organizations pertaining to dentistry; dealers and manufacturers of dental goods, to send to me at their earliest convenience, the necessary information (such as they may have) to complete a work like the one here proposed. Any other information which they may think of value to the Directory not herein referred to, will be thankfully received.

I earnestly request every dentist in the world to send to me his or her printed or plainly written professional card containing full address, and to briefly answer the following questions:

1. What is your native tongue, and with what languages are you conversant?

2. What colleges or institutions have you attended, and what degrees have you taken?

3. Of what societies, if any, are you a member?

4. What office, if any, have you held in either?

It will be evident to all that to complete this work will require considerable time and money, both of which have already been largely expended.

I have secured copyright for the book, and it will be sold by subscription. All subscribers who send in their money before November 1st, 1880, will have their names printed in **heavy letters,** and to such addresses will most likely be sent circulars, papers, books, samples, etc., setting forth all that is new and useful to the dental profession. In order to secure your name in the Directory, you will please send the desired information *without delay*, and state whether you would like to become a subscriber.

The price of the book will be five dollars. You are not required to purchase it in order to have your name inserted.

All dental journals in the world will please copy this article for the good of the profession at large, and send me a copy. We will then give your journal ample notice in the Directory.

Hoping that this will receive favorable and prompt attention, I am Very respectfully,

B. M. Wilkerson, M. D., D. D. S.,
Junior Editor and Proprietor of "The Independent Practitioner,"
68 N. Charles Street, Baltimore, Md.,
United States of America.

The enterprise indicated by the above communication is one of importance and interest to every dentist of this country, and to those of other countries as well.

This as it seems, is not to be a complete history of dentistry but a full reference work in respect to persons and things.

This is a work that cannot be accomplished without the hearty co-operation on the part of the profession, no man unaided can do this work as it should be done, so then let every one give his modicum of help. Such a work complete and accurate would find a possessor in every dentist in the world—who speaks the English language at least—who has any professional aspirations at all.

Such a work as this, followed by a complete history of the profession, would constitute an invaluable addition to the special literature of dentistry, and it is to be hoped that ere long this want will be supplied; it will if the profession give the proper aid and co operation; the which let every one do according to his ability.

